# Managing cardiovascular risk factors in patients with chronic kidney disease: pharmacological and non-pharmacological interventions in the Copenhagen CKD Cohort

**DOI:** 10.1093/ckj/sfae158

**Published:** 2024-06-13

**Authors:** Ellen Linnea Freese Ballegaard, Nicholas Carlson, Morten Buus Jørgensen, Ida Maria Hjelm Sørensen, Helene Trankjær, Anna Birna Almarsdóttir, Susanne Bro, Bo Feldt-Rasmussen, Anne-Lise Kamper, for The Copenhagen CKD Cohort Study Group

**Affiliations:** Department of Nephrology, Rigshospitalet, University of Copenhagen, Copenhagen, Denmark; Department of Clinical Medicine, Copenhagen University, Copenhagen, Denmark; Department of Nephrology, Rigshospitalet, University of Copenhagen, Copenhagen, Denmark; Department of Nephrology, Rigshospitalet, University of Copenhagen, Copenhagen, Denmark; Department of Nephrology, Rigshospitalet, University of Copenhagen, Copenhagen, Denmark; Department of Nephrology, Rigshospitalet, University of Copenhagen, Copenhagen, Denmark; Department of Pharmacy, Faculty of Health and Medical Sciences, University of Copenhagen, Copenhagen, Denmark; Department of Nephrology, Rigshospitalet, University of Copenhagen, Copenhagen, Denmark; Department of Nephrology, Rigshospitalet, University of Copenhagen, Copenhagen, Denmark; Department of Clinical Medicine, Copenhagen University, Copenhagen, Denmark; Department of Nephrology, Rigshospitalet, University of Copenhagen, Copenhagen, Denmark

**Keywords:** adherence, blood pressure, cardiovascular, chronic kidney disease, dyslipidemia

## Abstract

**Background:**

Although cardiovascular morbidity and mortality are substantial in patients with chronic kidney disease (CKD), guideline-directed treatment of cardiovascular risk factors remains a challenge.

**Methods:**

Observational, cross-sectional study including patients aged 30–75 years with CKD stage 1–5 without kidney replacement therapy from a tertiary hospital outpatient clinic. Data were obtained through patient interview, clinical examination, biochemical work-up, and evaluation of medical records and prescription redemptions. Guideline-directed treatment was evaluated as pharmacological interventions: antihypertensive and lipid-lowering therapy including adverse effects and adherence estimated as medication possession ratio (MPR); and non-pharmacological interventions: smoking status, alcohol consumption, body mass index (BMI), and physical activity.

**Results:**

The cohort comprised 741 patients, mean age 58 years, 61.4% male, 50.6% CKD stage 3, 61.0% office blood pressure ≤140/90 mmHg. Antihypertensives were prescribed to 87.0%, median number of medications 2 (IQR 1;3), 70.1% received renin–angiotensin system inhibition, 25.9% reported adverse effects. Non-adherence (MPR < 80%) was present in 23.4% and associated with elevated blood pressure (OR 1.53 (95% CI 1.03;2.27)) and increased urinary albumin excretion, *P* < 0.001. Lipid-lowering treatment was prescribed to 54.0% of eligible patients, 11.1% reported adverse effects, and 28.5% were non-adherent, which was associated with higher LDL cholesterol, *P* = 0.036. Overall, 19.2% were current smokers, 16.7% overconsumed alcohol according to Danish health authority recommendations 69.3% had BMI ≥ 25 kg/m^2^, and 38.3% were physically active <4 hours/week. Among patients prescribed antihypertensives, 51.9% reported having received advice on non-pharmacological interventions.

**Conclusions:**

Improved management of cardiovascular risk in patients with CKD entails intensified medical treatment and increased focus on patient adherence and non-pharmacological interventions.

## Graphical Abstract

**Graphical Abstract:**
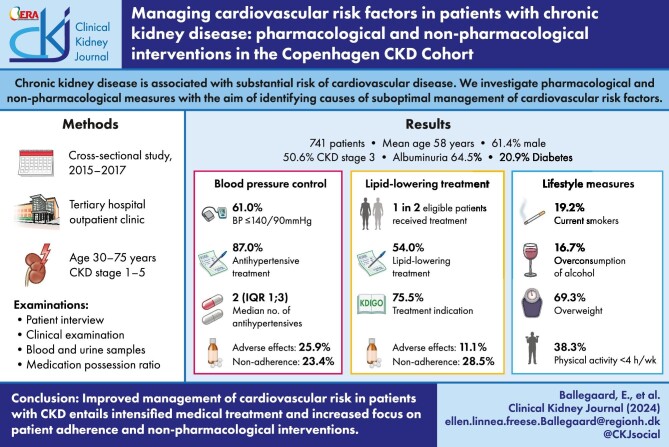

KEY LEARNING POINTS **What was known**:

- Cardiovascular morbidity and mortality are substantial in patients with chronic kidney disease (CKD).
- In CKD, risk factors for cardiovascular disease include both traditional and kidney-related risk factors.
- Observational data suggest guideline-directed management of cardiovascular risk in CKD is insufficient.

**This study adds**:

- Patient non-adherence and body mass index ≥25 kg/m^2^ were associated with office blood pressure >140/90 mmHg but not with a higher number of antihypertensives or more patient-reported adverse effects compared with office blood pressure ≤140/90 mmHg.
- Lipid-lowering therapy was only prescribed in half of eligible patients and >25% of treated patients were non-adherent to treatment.
- Although prevalence rates of smoking, excessive alcohol consumption, overweight, and physical inactivity were substantial, only half of patients on antihypertensive medication reported receiving information on non-pharmacological interventions.

**Potential impact**:

- Antihypertensive and lipid-lowering treatment should be intensified.
- Non-adherence is an important factor in not achieving treatment goals and should be addressed.
- More focus needs to be directed toward advice on non-pharmacological interventions.

## INTRODUCTION

Chronic kidney disease (CKD) induces a substantial risk of cardiovascular morbidity and mortality driven by a combination of traditional risk factors, such as hypertension and dyslipidemia, and factors related to kidney insufficiency, such as alterations of the calcium-phosphate metabolism, inflammation, oxidative stress, endothelial- and platelet dysfunction, and electrolyte imbalance [1, 2]. Hypertension is, furthermore, associated with increased risk of progression to end-stage kidney disease [3].

Pharmacological and non-pharmacological interventions aiming to ensure adequate blood pressure (BP) control in patients with CKD are advocated in all guidelines, although treatment goals are diverse (Supplementary Table S1) [4–8]. Pharmacological recommendations include inhibition of the renin–angiotensin-system (RAS) in patients with albuminuria [4] and non-pharmacological recommendations comprise general advice on healthy lifestyle, including salt restriction [4–6]. Lipid-lowering treatment is suggested or recommended in specific CKD subgroups irrespective of plasma lipid levels [9].

Observational studies have repeatedly shown poor office BP control in patients with CKD [10–22]. Suboptimal medication adherence [23], arterial stiffness [11, 15], and concerns related to adverse effects such as orthostatic hypotension [11] have been proposed as possible explanations. Other explanations may be insufficient use of pharmacological and/or non-pharmacological antihypertensive treatment by nephrologists. Data on the use of lipid-lowering treatment in CKD remain limited.

The Copenhagen CKD Cohort was established in 2015 to study cardiovascular morbidity in patients with CKD stages 1–5 without kidney replacement therapy (KRT) [24]. Based on qualitative and quantitative data, we investigate management of CKD with the aim of identifying suboptimal management of cardiovascular risk factors to help improve pharmacological and non-pharmacological care of patients with CKD.

## MATERIALS AND METHODS

### Study population

Details related to the Copenhagen CKD Cohort have been described previously [24]. Briefly, in an observational research study design, patients aged 30–75 years with any diagnosis of CKD stage 1–5 without KRT were consecutively recruited at a tertiary hospital outpatient clinic (Department of Nephrology, Rigshospitalet, Copenhagen, Denmark) from 2015 to 2017. The clinic provides highly specialized nephrological treatment to ∼4000 patients and serves regional and national functions. To ensure adequate time for implementation of guideline recommended treatments, patients had to have at least 6 months affiliation with the clinic. Exclusion criteria were previous kidney transplantation with a functioning graft, active malignancy, and pregnancy. Patients underwent extensive clinical and laboratory work-up following inclusion. Medical history and information on lifestyle were assessed from an interview-based standardized questionnaire (provided in the Supplemental Material) and existing medical records. Height, weight, and waist circumference were recorded. Body mass index (BMI) ≥25 kg/m^2^ defined overweight. Estimated glomerular filtration rate (eGFR) was calculated according to the creatinine-based Chronic Kidney Disease Epidemiology Collaboration (CKD-EPI) equation [25]. Albumin was measured in 24 h urine collections, and excretion >30 mg/24 h was defined as albuminuria [26].

### Blood pressure measurements

Office BP was measured in upright sitting position in a calm environment with sufficient support of the arm. After 5 minutes of rest, BP was measured on one arm with an oscillometric device (Microlife BP A3 Plus) using appropriate cuff size according to arm circumference. BP level was calculated as mean of the last two of three measurements.

To investigate the prevalence of masked and white-coat hypertension, a consecutive subgroup of patients was invited for a supplementary 24-hour ambulatory blood pressure monitoring (ABPM). The SpaceLab 90217–15Q recorders measured BP by the oscillometric method every 15 and 30 minutes during the day (8 a.m.–11 p.m.) and nighttime (11 p.m.–8 a.m.), respectively. Cuff width was selected according to arm circumference and applied on the non-dominant arm. Calculation of mean BP was based on a minimum of 14 daytime and seven nighttime recordings. Physical activity was not quantified. Daytime BP < 135/85 mmHg and nocturnal BP < 120/70 mmHg were considered normal [27]. Masked hypertension was defined as office BP ≤ 140/90 mmHg, but elevated ABPM; and white-coat hypertension as office BP > 140/90 mmHg, but normal ABPM.

Orthostatic hypotension was assessed in all patients with measurement of BP and heart rate after a 5-minute rest in supine position, and then 1 and 5 minutes after change to upright position. Orthostatic hypotension was defined as a fall in systolic BP ≥ 20 mmHg when changing position.

### Evaluation of medication adherence

Information about prescribed medication was obtained from the electronic prescription system, www.fmk-online.dk. This system holds information about all prescribed medications to Danish residents including details relating to redemption of prescriptions (medication name, prescribed dosage, dispensation date, and medicine dispensed).

#### Medication possession ratio

Medication possession ratio (MPR) was calculated as the proportion of daily doses covered during a period of three prescription redemptions around the time of the clinical visit (Supplementary Figure S1). The number of oral dosing units (i.e. tablets, capsules) dispensed was calculated from the first two prescription redemptions before the clinical visit, while the period was calculated as the number of days between the first of these prescription redemptions and the first prescription redemptions after the clinical visit. This is a well-established method of calculating MPR [28]. If medication doses were changed or the prescription discontinued within this period, it was considered in the calculation.

Patients included in the analysis had at least 4 months of follow-up after the clinical visit. End of follow-up was 1 March 2017. Prescriptions categorized as ‘pro necessitate’ were omitted from the analysis, as well as apparently discontinued prescriptions (patient declined receiving the medication and MPR < 40%, *n* = 11). Patients who had stored medication for another 6 months use were omitted from the analysis.

### Study outcomes

Details of study outcomes are provided in Table 1. Pharmacological interventions were evaluated as proportion of patients achieving guideline recommendations for BP targets (including ABPM), treatment with RAS inhibition, lipid-lowering treatment, adherence, and tolerance to treatment (i.e. adverse effects and orthostatic hypotension). Evaluation of BP included both the 2012 KDIGO goal of BP ≤ 140/90 mmHg in patients without albuminuria and BP ≤ 130/80 mmHg in patients with albuminuria, which were the actual treatment goals at the time of the study, and the 2021 KDIGO goal of systolic BP < 120 mmHg. Lipid-lowering treatment was evaluated in accordance with the 2013 KDIGO Clinical Practice Guideline for Lipid Management. Non-adherence was defined as an MPR < 80% [29] Non-pharmacological intervention was evaluated as current smoking, overconsumption of alcohol (>7 and >14 standard units/week for women and men, respectively), BMI ≥ 25 kg/m^2^, increased waist circumference (>80 cm for women and >94 cm for men [30]), limited physical activity (<4 hours/week) [31], and proportion of patients informed of non-pharmacological interventions.

**Table 1:** Study outcomes.

**Pharmacological treatment**
*BP*
Proportion of patients with
*-* standardized office BP ≤ 140/90 mmHg in patients with urinary albumin excretion <30 mg/24 h and ≤130/80 mmHg in patients with urinary albumin excretion ≥30 mg/24 h^1^
- standardized office systolic BP < 120 mmHg^2^
-albuminuria and office BP > 130/80 mmHg receiving RAS inhibition
- orthostatic hypotension
- white-coat hypertension
- masked hypertension
- MPR < 80% for or self-reported non-adherence
- self-reported adverse effects
*Lipids*
Proportion of patients who
- were aged ≥50 years or aged <50 years with cardiovascular risk factors^4^ receiving lipid-lowering treatment^5^
- had MPR < 80% or self-reported non-adherence
- had self-reported current adverse effects or adverse effects that led to discontinuation of lipid-lowering treatment
**Non-pharmacological interventions**
Proportion of patients who
- currently smoked
- had alcohol consumption above Danish health authority recommendations^3^
- had BMI ≥ 25 kg/m^2^
- had waist circumference >80 cm for women and >94 cm for men (30)
- did weekly physical activity <4 hours/week (31)
- were informed about non-pharmacological interventions

MPR was calculated as the proportion of daily doses covered during a period of three prescription redemptions around the time for the clinical visit.

1 2012 KDIGO clinical practice guideline for the management of BP in CKD (4)

2 2021 KDIGO clinical practice guideline for the management of BP in CKD (8)

3 Women, >7 standard units/week; men, >14 standard units/week

4 Coronary disease, diabetes mellitus, prior ischemic stroke, or an estimated 10-year risk of coronary death or non-fatal myocardial infarction >10%

5 2013 KDIGO clinical practice guideline for lipid management in CKD (9)

### Statistical analyses

Categorical variables are presented with frequency distributions with percentages and compared using the *χ*^2^-test. Depending on normal distribution, continuous variables are presented as mean ± standard deviation (SD) or median and interquartile range (IQR) and compared with Student's *t*-test and one way-ANOVA or Mann–Whitney *U*-test and Kruskal–Wallis test. The association between adherence and BP control was analyzed in a univariate logistic regression model and a multivariate logistic regression model adjusted for age, sex, and number of antihypertensive medications. Patients with missing data on adherence were excluded in the logistic regression models. *P* ≤ 0.05 was considered significant. Statistical analyses were performed using R version 3.6.1.

### Ethics approval and reporting guidelines

The study protocol was approved by the Regional Committee on Health Research Ethics (H-3–2011–069) and the Danish Data Protection Agency. All participants signed a written informed consent prior to inclusion, For preparation of this paper, the STROBE (STrengthening the Reporting of OBservational studies in Epidemiology) cohort reporting guidelines were used [32].

## RESULTS

A total of 741 patients were included in the study. Mean age was 58 years, 61.4% were male, 20.9% had diabetes, median eGFR was 41 ml/min/1.73 m^2^ (IQR 29;59 ml/min/1.73 m^2^), and most patients had CKD stage 3 (50.6%). Patient characteristics are provided in Table 2.

**Table 2:** Demographic, clinical, and laboratory characteristics of participants in the Copenhagen CKD Cohort (*n* = 741).


Age, years	58 [13]
Male sex	455 (61.4)
Etiology of CKD	
Diabetic kidney disease	88 (11.9)
Vascular disease	29 (3.9)
Chronic tubular interstitial nephropathy	12 (1.6)
Chronic glomerulonephritis	206 (27.8)
Adult polycystic kidney disease	82 (11.1)
Other	121 (16.3)
Unknown	203 (27.4)
eGFR, ml/min/1.73 m^2^	46 [25]
CKD stages	
CKD 1 (eGFR > 90 ml/min/1.73 m^2^)	62 (8.4)
CKD2 (eGFR 60–90 ml/min/1.73 m^2^)	115 (15.5)
CKD3 (eGFR 30–59 ml/min/1.73 m^2^)	375 (50.6)
CKD4 (eGFR 15–29 ml/min/1.73 m^2^)	146 (19.7)
CKD5 (eGFR < 15 ml/min/1.73 m^2^)	43 (5.8)
Urinary albumin excretion	
Normal to mildly increased (<30 mg/day)	250 (35.5)
Moderately increased (30–300 mg/day)	256 (36.3)
Severely increased (>300–700 mg/day)	98 (13.9)
Very severely increased (>700–2200 mg/day)	84 (11.9)
Nephrotic range albuminuria (>2200 mg/day)	17 (2.4)
Comorbidity	
Diabetes mellitus type 1	17 (2.3)
Diabetes mellitus type 2	138 (18.6)
Stroke	69 (9.3)
Congestive heart failure	79 (10.7)
Coronary artery disease^a^	78 (10.5)
Atrial fibrillation or flutter	55 (7.4)
Chronic lung disease	72 (9.7)
Chronic liver disease	20 (2.7)
Chronic rheumatologic disease	160 (21.6)
Hyper-/hypothyroidism	44 (5.9)
Peripheral arterial disease	42 (5.7)

Values are mean [SD], median {IQR} or *n* (%).

a Coronary artery disease: angina pectoris, myocardial infarction, percutaneous coronary intervention, and/or bypass surgery.

### Blood pressure

Mean office systolic BP was 132.3 mmHg (SD 17.9) and mean diastolic BP was 80.9 mmHg (SD 11.4). Office BP ≤ 140/90 mmHg was recorded in 61.0% of patients, while 23.2% met the 2021 KDIGO recommendation of systolic BP < 120 mmHg (characteristics provided in Supplementary Table S2). Table 3 presents characteristics of patients with BP ≤ 140/90 mmHg and BP > 140/90 mmHg. Among patients with BP > 140/90 mmHg, 22 patients (7.6%) had white-coat hypertension according to previous ABPM or were within the limits of an individually defined higher BP goal. The frequency of patients with BP > 140/90 mmHg increased with stage of kidney failure (Supplementary Table S3).

**Table 3:** Characteristics of patients on antihypertensive treatment with and without BP control (*n* = 645).

	Controlled BP ≤ 140/90 mmHg *n* = 387	Uncontrolled BP > 140/90 mmHg *n* = 258	*P*
Age, years	58 [13]	60 [12]	0.005
Male sex	215 (55.6)	184 (71.3)	<0.0001
Systolic BP (mmHg)	122.3 [11.2]	148.4 [14.3]	<0.0001
Diastolic BP (mmHg)	76.1 [8.8]	87.8 [11]	<0.0001
eGFR (ml/min/1.73 m^2^)	46 [23]	42 [23]	0.020
Urinary albumin excretion (mg/24 h)	68 {16–312}	126 {22–601}	0.004
Diabetes mellitus	84 (21.7)	68 (26.5)	0.20
Coronary artery disease	48 (12.4)	25 (9.7)	0.35
Pharmacological treatment			
Number of antihypertensives	2 {1;3}	2 {2;3}	0.071
RAS inhibitor	284 (73.4)	168 (65.1)	0.031
Beta-blocker	142 (36.7)	128 (49.6)	0.001
Calcium channel blocker	161 (41.6)	122 (47.3)	0.18
Diuretics	250 (64.4)	175 (67.8)	0.45
Lifestyle parameters			
Overweight (BMI ≥ 25 kg/m^2^)	262 (67.7)	205 (79.8)	0.001
Obesity (BMI ≥ 30 kg/m^2^)	129 (33.3)	104 (40.5)	0.078
Increased waist circumference	304 (78.8)	215 (83.7)	0.15
Current smoker	78 (20.2)	51 (19.8)	0.98
Alcohol intake above recommended	63 (16.3)	46 (17.8)	0.68
Inactive/low physical activity level	160 (41.3)	103 (39.9)	0.78

Values are mean [SD], median {IQR} or *n* (%). *P* values for categorical variables are given by chi-square test; *P* values for continuous variables are given by *t-*test.

Coronary artery disease: angina pectoris, myocardial infarction, percutaneous coronary intervention, and/or bypass surgery.

Increased waist circumference: women: waist circumference >80 cm; men: waist circumference >94 cm Alcohol intake above recommended: women, >7 standard units/week; men, >14 standard units/week

Inactive/low physical activity level: <4 hours of light exercise/week.

Missing values: *n*, urinary albumin excretion, 34; BMI, 1; abdominal fat distribution, 2

Urinary albumin excretion was measured in 705 patients (95.1%), with albuminuria detected in 455 patients (64.5%). Of note, office BP > 130/80 mmHg was observed in 334 patients (73.4%) with albuminuria.

ABPM was accepted by 71 of 110 invited patients (64.5%) with a complete dataset available in 67 patients (9.0% of the entire cohort). Baseline characteristics remained comparable with exception of better kidney function in patients who underwent ABPM (Supplementary Table S4). Elevated daytime and/or nighttime BP was found in 46 patients (68.7%), masked hypertension was found in 24 patients (35.8%), while two patients (3.0%) had white-coat hypertension.

### Pharmacological interventions

#### Antihypertensive treatment

In total, 645 patients (87.0%) were prescribed antihypertensive medication, ranging from 64.5% of patients with CKD1 to 93.0% of patients with CKD5. The median number of antihypertensive medications was 2 (IQR 1;3), with >40% treated with ≥3 antihypertensive medications (1, 26.4%; 2, 30.4%; 3, 28.7%; ≥4, 14.6%). RAS inhibitors (angiotensin-receptor blockers or angiotensin-converting enzyme inhibitors) were prescribed in 452 patients (70.1%) receiving antihypertensive medication, with concomitant diuretic treatment in 290 patients (45.0%). Among the 455 patients with albuminuria, 62.6% were prescribed RAS inhibitors, while 60.8% of the 334 patients with albuminuria and BP > 130/80 mmHg were prescribed RAS inhibitors. Reasons for non-treatment in patients with albuminuria and BP > 130/80 mmHg included prior treatment-associated increase in plasma creatinine and/or plasma potassium (23.7%), prior patient-related side effects (9.9%), renal artery stenosis (5.3%), and other specified reasons (4.6%). Importantly, no specific reason was provided in 56.6% of patients.

##### Adverse effects and orthostatic hypotension

Adverse effects to antihypertensive medications were reported by 167 patients (25.9%) (data missing, *n* = 58). The number of self-reported adverse effects remained unchanged between patients irrespective of BP control, adherence, and CKD stage. Orthostatic hypotension was demonstrated in 165 patients (22.3%) in the entire cohort. Prevalence of orthostatic hypotension remained comparable between patients with and without BP control (24.8% vs. 21.2%, *P* = 0.34).

##### Adherence to treatment

**Figure 1:**
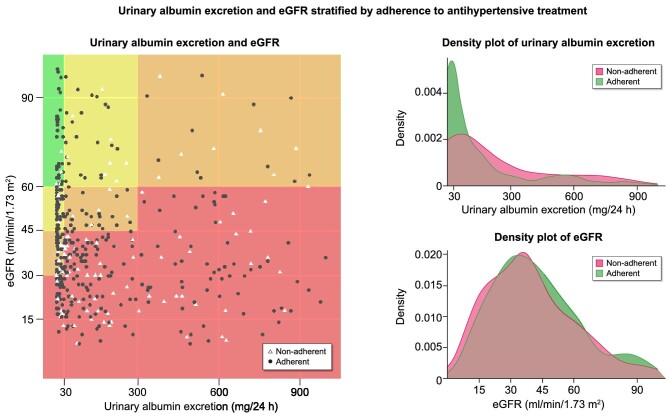
Abbreviations: eGFR, estimated glomerular filtration rate. Adherence was evaluated in 561 patients. Non-adherence to antihypertensive treatment was defined as medication possession ratio <80%. Urinary albumin excretion was estimated in 24h urine collections. Estimated glomerular filtration rate was calculated with the CKD-EPI equation. Colors in the left-hand figure are inspired by the KDIGO risk classification in patients with chronic kidney disease.

MPR was evaluated in 561 patients (86.7%). Non-adherence (MPR < 80%) was demonstrated in 131 patients (23.4%). Of note, 69.6% of adherent patients and 56.9% of non-adherent patients reported never forgetting to take their antihypertensive medication (*P* = 0.042). Non-adherence was associated with a BP > 140/90 mmHg in both a univariate model [odds ratio (OR) 1.53 (95% CI 1.03;2.27, *P* = 0.033)] and adjusted for age, sex, and number of antihypertensive medications [OR 1.57 (95% CI 1.05;2.35, *P* = 0.029)]. The association of non-adherence to antihypertensive treatment, eGFR, and albuminuria is shown in Fig. 1, demonstrating similar eGFR (*P* = 0.12), but increased urinary albumin excretion (*P* < 0.001) in non-adherent patients compared with adherent patients.

#### Lipid-lowering treatment

Lipid-lowering treatment was indicated in 561 patients (75.7%) according to the KDIGO CKD guideline [9], with 303 patients (54.0%) treated. An additional 21 patients received treatment without meeting guideline criteria. Of note, treatment was prescribed to 293 of 528 patients (55.5%) ≥50 years and to 10 out of 33 patients (30.3%) <50 years with known cardiovascular risk factors. No significant differences were observed in the proportion of eligible patients treated between CKD stages.

##### Adverse effects

Adverse effects were reported by 37 patients (11.1%) (missing data, *n* = 13) with no difference in frequency between adherent and non-adherent patients (data not shown), nor between stages of CKD (missing data, *n* = 13). Previous prescription of lipid-lowering treatment was reported by 69 patients, with 20 patients (29.0%) previously discontinuing treatment due to adverse events.

##### Adherence to treatment

MPR was evaluated in 267 patients (79.9%), with 28.5% non-adherent to treatment and no significant difference between stages of CKD. Non-adherence was associated with increased plasma LDL cholesterol (2.7 mmol/l vs. 2.4 mmol/l, *P* = 0.036). There were no differences in plasma levels of total cholesterol (4.8 vs. 4.5 mmol/l, *P* = 0.083), HDL-cholesterol (1.4 vs. 1.5 mmol/l, *P* = 0.40), or triglycerides (2.1 vs. 1.9 mmol/l, *P* = 0.20) between groups.

### Non-pharmacological interventions

Data on non-pharmacological interventions are provided in Table 4. Overall, 142 patients (19.2%) were current smokers; 124 patients (16.7%) had an overconsumption of alcohol; 513 patients (69.3%) and 248 patients (33.5%) were overweight (BMI ≥ 25 kg/m^2^) and obese (BMI ≥ 30 kg/m^2^), respectively; 578 patients (78.2%) had increased waist circumference; and 284 patients (38.3%) were physically active < 4 hours per week. Patients with more progressed CKD were more likely to be overweight with increased waist circumference and low level of physical activity.

**Table 4:** Lifestyle parameters in the Copenhagen CKD Cohort (*n* = 741).

Weight and fat distribution	
BMI (kg/m^2^)	28.5 [5.9]
Overweight (BMI ≥ 25 kg/m^2^)	513 (69.3)
Obesity (BMI ≥ 30 kg/m^2^)	248 (33.5)
Waist circumference (cm)	101.8 [16.4]
Increased waist circumference^a^	578 (78.2)
Smoking status	
Never	302 (40.8)
Former	297 (40.1)
Current	142 (19.2)
Smoking pack years^b^	5 {0–24}
Alcohol intake	
Units/week	2 {0–7}
Above recommended^c^	124 (16.7)
Physical activity^d^	
Inactive	116 (15.7)
Low	168 (22.7)
Moderate	379 (51.1)
Vigorous	78 (10.5)
Self-reported dietary awareness of	
Salt intake	465 (62.8)
Calorie intake	426 (57.7)
Protein intake	285 (38.5)

Values are mean [SD], median {IQR} or *n* (%).

a Increased waist circumference: women, waist circumference >80 cm; men, waist circumference >94 cm

b One pack year was defined as 20 cigarettes (or an amount of tobacco corresponding the amount in 20 cigarettes) per day for 1 year.

^c^Alcohol intake above recommended: women, >7 standard units/week; men, >14 standard units/week

d Inactive, almost totally inactive or light physical activity <2 hours/week; low, light physical activity for 2–4 hours/week; moderate, light physical activity >4 hours per week or strenuous physical activity for 2–4 hours per week; vigorous, strenuous physical activity for >4 hours/week

Among patients on antihypertensive medication, 335 patients (51.9%) reported having received information on non-pharmacological interventions related to treatment of hypertension (data missing, *n* = 56). No difference was noted between patients with (52.2%) and without BP control (51.6%), *P* = 0.55. BMI ≥ 25 kg/m^2^ was associated with BP > 140/90 mmHg while there was no association with other lifestyle parameters on BP control. Awareness of salt-, calorie-, and protein intakes were reported by 416 patients (64.5%), 367 patients (57.2%), and 249 patients (38.6%), respectively, with no difference between patients with and without BP control.

## DISCUSSION

In a comprehensive dataset including 741 outpatients with CKD without KRT we found that appropriate guideline-directed RAS inhibition and lipid-lowering treatment were insufficiently used, and a substantial proportion of patients failed to reach recommended BP targets, with non-pharmacological parameters and non-adherence partially accountable.

BP control is an important factor in the management of CKD as advocated in several guidelines. However, both in our cohort and in other national cohorts of patients with CKD without KRT [10, 12–16, 18–22], reaching treatment targets remain difficult. This is in contrast to the clinical trials on intensive antihypertensive treatment in CKD populations (AASK and SPRINT) where a mean BP well below 130/80 mmHg was obtained with 3.0 antihypertensives [33, 34]. In our cohort, a median of 2 (IQR 1;3) antihypertensives were prescribed. As such, intensified pharmacological intervention could appear possible, albeit conventional (BP ≤ 140/90 mmHg) and/or restrictive BP targets (systolic BP < 120 mmHg) have proved challenging to reach in a clinical setting.

RAS inhibition was prescribed in 70.1% of patients in our cohort and appeared to be insufficiently used in patients with albuminuria with treatment only in 62.6% of patients. In other CKD cohorts, RAS inhibition has been reported in 74–83% of patients on antihypertensive treatment [10, 13, 18, 15]. Pre-existing information detailing the cause of non-use remains limited, and our data only demonstrated specific reasoning for lack of appropriate treatment in approximately half of non-treated patients with albuminuria. As in the German CKD cohort [9], use of appropriate lipid-lowering treatment was suboptimal in our cohort, with treatment prescribed to only one in two eligible patients. In the German CKD cohort, it was speculated that the undertreatment could be explained by discontinuation of lipid-lowering therapy due to side effects [35]. We found this to be the case in only one-third of previously treated patients leaving a large percentage with no apparent reason for non-treatment.

Patient adherence to treatment remains a challenge. Studies from general populations demonstrate adherence to antihypertensive and lipid-lowering therapy down to 50% after 1 year of treatment [29, 36, 37]. We found approximately one-quarter of patients to be non-adherent to treatment that was associated with poor BP control, higher grade of albuminuria, and higher level of LDL cholesterol. Patient involvement and education on the necessity of pharmacological treatment play an important role in addressing non-adherence to medicines [38]. It is noteworthy that half of the non-adherent patients claimed to never forget their antihypertensive medication, despite the MPR showing otherwise. However, social desirability bias might play a role in the high self-reported adherence estimated with a non-validated tool. Non-adherence to antihypertensive treatment in other CKD populations has been reported in around one-third of patients [23, 39, 40]. Furthermore, an American cohort demonstrated proportional decreased adherence to antihypertensive treatment with increasing stage of CKD [23]. In our data, patients with CKD5 were the least adherent to antihypertensive treatment, but the most adherent to lipid-lowering treatment. Among patients with CKD, the choice of withholding treatment might be influenced by concerns about polypharmacy and adverse effects, doubts on efficacy of the prescribed drugs, and poor communication with the physician [41]. Studies on prevalence of adverse effects in CKD remain limited, with prevalence of adverse effects related to any medication previously reported to be 58% [42]. In this study, 11.1% and 25.9% reported adverse effects to lipid-lowering and antihypertensive therapy, respectively. Furthermore, report of adverse effects was not related to non-adherence or absence of BP control, and physician's decision to withhold guideline-directed treatment was only partly explained by presence of previous adverse effects. In general, non-pharmacological interventions remained insufficiently implemented in our cohort. The prevalence of overweight and physical inactivity was high, with rates increasing with higher CKD stage, while overall awareness of diet recommendations was low. Overweight and obesity in CKD seem to be a global issue with prevalence rates of 77%–83% reported in prior CKD cohorts [10, 15, 16, 43]. Importantly, we found that overweight was associated with insufficient BP control, underscoring the potential of non-pharmacological intervention on hypertension in CKD. However, as also demonstrated in the CKD Outcomes and Practice Patterns Study [44] counseling on non-pharmacological interventions affirmed by patients in our cohort was deficient irrespective of CKD stage deficient.

### Strengths and limitations

The strength of the study lays in the detailed individual work-up in all cohort participants, including report of adverse effects and lifestyle, study of medical records, as well as medication adherence evaluated by dispensed medication using a nationwide electronic prescription system.

A number of limitations apply. First, BP control was defined based on measurement of office BP at a single visit, and ABPM was limited to a random subsample, with results suggesting overestimation of BP control and substantial prevalence of masked hypertension. Second, the study questionnaire was not based on validated tools for evaluation of adherence or non-pharmacological interventions. Third, datasets were incomplete in 60 patients (9%). Of note, details regarding the individual patient affiliation with the outpatient clinic, patient-doctor continuity, and non-pharmacological interventions delivered by other health care providers (i.e. dieticians and physiotherapists) remained unregistered. Similarly, no formal evaluation of clinician awareness of guideline recommendations was performed. Nonetheless, we consider 6 months affiliation with a highly specialized outpatient clinic sufficient time for implementation of guideline-directed management. These limitations should be considered when interpreting the use of our results in a clinical context.

In conclusion, our data demonstrate insufficient implementation of appropriate guideline-directed BP control, RAS inhibition, and lipid-lowering treatment with non-pharmacological parameters and non-adherence partially accountable.

## Supplementary Material

sfae158_Supplemental_File

## Data Availability

The data underlying this article cannot be shared publicly due to Danish legal restrictions. The data will be shared on reasonable request to the corresponding author, provided relevant ethical and legal permissions have been attained previously, and researchers meet the criteria for access to confidential data.
