# Supplementary material for: Managing cardiovascular risk factors in patients with chronic kidney disease: pharmacological and non-pharmacological interventions in the Copenhagen CKD Cohort
Source: Clin Kidney J. 2024 Jun 13;17(7):sfae158. doi: 10.1093/ckj/sfae158 (PMC11229031; doi:10.1093/ckj/sfae158)
Supplement: sfae158_Supplemental_File [file sfae158_supplemental_file.docx]

# Supplemental material

Supplementary Table S1: Guideline recommendations of target blood pressure in patients with chronic kidney disease

Supplementary Table S2: Comparison of patients with systolic blood pressure (BP) <120 mmHg and ≥120 mmHg

Supplementary Table S3: Comparison of patients across stages of CKD

Supplementary Table S4: Comparison of patients with and without ambulatory blood pressure measurement

Supplementary Figure S1: Estimation of medication possession ratio (MPR)

Selected sections of the original questionnaire translated from Danish to English

| **Supplementary Table S1: Guideline recommendations of target blood pressure in patients with chronic kidney disease** | |
| --- | --- |
| KDIGO 2012 | Without albuminuria: ≤140/90 mmHg  With albuminuria: ≤130/80 mmHg |
| ACC/AHA 2017 | <130/80 mmHg |
| ESC/ESH 2018 | Systolic blood pressure <130-139 mmHg |
| ISH 2020 | <130/80 mmHg |
| KDIGO 2021 | Systolic blood pressure <120 mmHg |
| KDIGO 2012, Kidney Disease: Improving Global Outcomes(4) ACC/AHA 2017, American College of Cardiology & American Heart Association(6) ESC/ESH 2018, European Society of Cardiology & European Society of Hypertension(5)  ISH 2020, International Society of Hypertension(7)  KDIGO 2021, Kidney Disease: Improving Global Outcomes(8) | |

| **Supplementary Table S2: Comparison of patients with systolic blood pressure <120 mmHg and ≥120 mmHg** | | | | |
| --- | --- | --- | --- | --- |
|  | **Systolic BP ≤120 mmHg**  **n=172** | **Systolic BP >120 mmHg**  **n=569** | **P** |  |
| Age, years | 54 [13.3] | 59 [12.7] | <0.0001 |  |
| Male sex | 66 (38.4) | 389 (68.4) | <0.0001 |  |
| Systolic blood pressure, mmHg | 110.2 [7.9] | 139.0 [14.4] | <0.0001 |  |
| Diastolic blood pressure, mmHg | 72.4 [9.3] | 83.5 [10.6] | <0.0001 |  |
| Estimated glomerular filtration rate, ml/min/1.73 m^2^ | 51 [25.4] | 45 [24.1] | 0.002 |  |
| Urinary albumin excretion, mg/24 h | 34 {7-161} | 100 {19-494} | <0.0001 |  |
| Diabetes mellitus | 32 (18.6) | 123 (21.7) | 0.451 |  |
| Coronary artery disease^1^ | 20 (11.6) | 58 (10.2) | 0.693 |  |
| Antihypertensive treatment | 145 (84.3) | 500 (87.9) | 0.275 |  |
| Lifestyle parameters |  |  |  |  |
| Overweight (BMI ≥25 kg/m^2^) | 101 (58.4) | 412 (72.5) | 0.001 |  |
| Abdominal fat distribution^2^ | 128 (74.4) | 450 (79.4) | 0.204 |  |
| Current smoker | 32 (18.6) | 110 (19.3) | 0.564 |  |
| Alcohol intake above recommended^3^ | 99 (17.4) | 25 (14.5) | 0.444 |  |
| Inactive/low physical activity level^4^ | 53 (30.8) | 231 (40.6) | 0.026 |  |
| Values are mean [SD], median {IQR} or n (%). P-values for categorical variables are given by Chi-square test; p-values for continuous variables are given by t-test.  Abbreviations: BMI, Body Mass Index; BP, blood pressure;  ^1^Coronary artery disease: angina pectoris, myocardial infarction, percutaneous coronary intervention and/or bypass surgery.  ^2^Abdominal fat distribution: women: waist circumference >80 cm; men waist circumference >94 cm ^3^Alcohol intake above recommended: women: >7 standard units/week; men: >14 standard units/week  ^4^Inactive/low physical activity level: <4 hours of light exercise/week.  Missing values, n: urinary albumin excretion: 34, BMI: 1, abdominal circumference: 2 | | | |  |

| **Supplementary Table S3: Comparison of patients across stages of CKD** | | | | | | | | | |
| --- | --- | --- | --- | --- | --- | --- | --- | --- | --- |
|  | | | **CKD 1 n=62** | **CKD 2 n=115** | **CKD 3  n=375** | **CKD 4 n=146** | **CKD 5 n=43** | **P** |  |
| Age, years | | | 44 [11] | 51 [13] | 61 [12] | 62 [11] | 61 [11] | <0.001 |  |
| Male sex | | | 36 (58.1) | 64 (55.7) | 242 (64.5) | 86 (58.9) | 27 (62.8) | 0.42 |  |
| Systolic blood pressure, mmHg | | | 127.7 [17.7] | 127.2 [13.7] | 133.6 [18.7] | 131.3 [17.6] | 143.8 [15.7] | <0.001 |  |
| Diastolic blood pressure, mmHg | | | 82.5 [12.2] | 82.4 [9.7] | 81.3 [11.5] | 77.9 [11.3] | 81.9 [12.2] | 0.007 |  |
| BP≤140/90 mmHg | | | 20 (32.3) | 33 (28.7) | 152 (40.5) | 54 (37.0) | 30 (69.8) | <0.001 |  |
| eGFR, ml/min/1.73 m^2^ | | | 101 [8] | 71 [9] | 43 [9] | 22 [4] | 11 [2] | <0.001 |  |
| Urinary albumin excretion, mg/24 h | | | 34 {8;165} | 39 {10;412} | 59 {11;299} | 126 {25;421} | 483 {154;985} | <0.001 |  |
| Diabetes mellitus | | | 1 (1.6) | 5 (4.4) | 90 (24.0) | 46 (31.5) | 13 (30.2) | <0.001 |  |
| Coronary artery disease | | | 1 (1.6) | 4 (3.5) | 49 (13.1) | 17 (11.6) | 7 (16.3) | 0.004 |  |
| **Pharmacological treatment** | | | | | | | | | |
| Antihypertensive treatment | | | 40 (64.5) | 94 (81.7) | 338 (90.1) | 133 (91.1) | 40 (93.0) | <0.001 |  |
|  | No. of antihypertensives | | 1 {1;2} | 2 {1;3} | 2 {2;3} | 2 {2;3} | 3 {2;3} | <0.001 |  |
|  | Adverse effects | | 15 (42.9) | 22 (25.9) | 94 (30.2) | 29 (24.6) | 7 (18.4) | 0.13 |  |
|  | | *Missing* | *5* | *9* | *27* | *15* | *2* |  |  |
|  | Non-adherence | | 6 (17.1) | 19 (23.5) | 67 (23.7) | 27 (22.0) | 12 (30.8) | 0.72 |  |
|  | | *Missing* | *5* | *13* | *55* | *10* | *1* |  |  |
| Lipid-lowering treatment | | | 9 (14.5) | 36 (31.3) | 188 (50.1) | 66 (45.2) | 25 (58.1) | <0.001 |  |
|  | Eligible patients treated | | 8 (38.1) | 30 (46.9) | 177 (56.7) | 64 (50.0) | 24 (66.7) | 0.11 |  |
|  | Adverse effects | | 3 (33.3) | 2 (6.1) | 25 (14.0) | 5 (7.7) | 2 (8.3) | 0.13 |  |
|  | | *Missing* | *0* | *3* | *10* | *1* | *1* |  |  |
|  | Non-adherence | | 3 (37.5) | 9 (31.0) | 36 (24.8) | 24 (38.7) | 4 (17.4) | 0.20 |  |
|  | | *Missing* | *1* | *7* | *43* | *4* | *2* |  |  |
| **Non-pharmacological treatment** | | | | | | | | | |
| Overweight (BMI≥25 kg/m^2^) | | | 27 (43.5) | 71 (61.7) | 278 (74.3) | 106 (72.6) | 31 (72.1) | <0.001 |  |
| Obesity (BMI≥30 kg/m^2^) | | | 15 (24.2) | 31 (27.0) | 131 (27.0) | 53 (36.3) | 18 (41.9) | 0.14 |  |
| Increased waist circumference | | | 34 (54.8) | 86 (75.4) | 303 (81.0) | 119 (81.5) | 36 (83.7) | <0.001 |  |
| Current smoker | | | 12 (19.4) | 21 (18.3) | 74 (19.7) | 28 (19.2) | 7 (16.3) | 0.98 |  |
| Alcohol intake above recommended | | | 9 (14.5) | 23 (20.0) | 69 (18.4) | 17 (11.6) | 6 (14.0) | 0.31 |  |
| Inactive/low physical activity level | | | 12 (19.4) | 31 (27.0) | 138 (36.8) | 77 (52.7) | 26 (60.5) | <0.001 |  |
| Received information^1^ | | | 16 (45.7) | 49 (57.6) | 188 (60.3) | 62 (52.1) | 20 (52.6) | 0.33 |  |
|  | | *Missing* | *5* | *9* | *26* | *14* | *2* |  |  |
| Abbreviations: BMI, body mass index; BP, blood pressure; eGFR, estimated glomerular filtration rate.  Values are mean [SD], median {IQR} or n (%). P-values for categorical variables are given by Chi-square test; p-values for continuous variables are given by Kruskal-Wallis test or one-way ANOVA according to normal distribution.  ^1^Only patients on antihypertensive treatment were asked this question  Coronary artery disease: angina pectoris, myocardial infarction, percutaneous coronary intervention and/or bypass surgery.  Increased waist circumference: women: waist circumference >80 cm; men: waist circumference >94 cm  Alcohol intake above recommended: Women:>7 standard units/week; men: >14 standard units/week  Inactive/low physical activity level: <4 hours of light exercise/week.  Missing values, n: urinary albumin excretion: 34, BMI: 1, abdominal fat distribution: 2 | | | | | | | | | |

| **Table S4: Comparison of patients with and without ambulatory blood pressure measurement** | | | | |
| --- | --- | --- | --- | --- |
|  | **ABPM  n=67** | **No ABPM  n=674** | **P** |  |
| Age, years | 55 [15] | 58 [13] | 0.11 |  |
| Male sex | 40 (59.7) | 415 (61.6) | 0.87 |  |
| Systolic blood pressure, mmHg | 130.7 [16.4] | 132.4 [18.1] | 0.45 |  |
| Diastolic blood pressure, mmHg | 81.8 [10.8] | 80.9 [11.4] | 0.54 |  |
| BP ≤140/90 mmHg | 44 (65.7) | 408 (60.5) | 0.49 |  |
| Antihypertensive treatment | 55 (82.1) | 590 (87.5) | 0.28 |  |
| eGFR, ml/min/1.73 m^2^ | 53 [25] | 46 [24] | 0.030 |  |
| Urinary albumin excretion, mg/24 h | 26 {6-142} | 86 {18-457} | <0.001 |  |
| Diabetes mellitus | 11 (16.4) | 144 (21.4) | 0.43 |  |
| Coronary artery disease | 4 (6.0) | 74 (11.0) | 0.29 |  |
| Lifestyle parameters |  |  |  |  |
| Overweight (BMI≥25 kg/m^2^) | 45 (67.2) | 468 (69.5) | 0.79 |  |
| Obesity (BMI≥30 kg/m^2^) | 20 (29.9) | 228 (33.9) | 0.60 |  |
| Increased waist circumference | 51 (76.1) | 527 (78.4) | 0.78 |  |
| Current smoker | 6 (9.0) | 136 (20.2) | 0.039 |  |
| Alcohol intake above recommended | 16 (23.9) | 108 (16.0) | 0.14 |  |
| Inactive/low physical activity level | 23 (34.3) | 261 (38.7) | 0.57 |  |
| Values are mean [SD], median {IQR} or n (%). P-values for categorical variables are given by Chi-square test; p-values for continuous variables are given by t-test.  Abbreviations: ABPM, ambulatory blood pressure measurement; BMI, body mass index; BP, blood pressure; eGFR, estimated glomerular filtration rate.  Coronary artery disease: angina pectoris, myocardial infarction, percutaneous coronary intervention and/or bypass surgery.  Increased waist circumference: women: waist circumference >80 cm; men: waist circumference >94 cm  Alcohol intake above recommended: Women:>7 standard units/week; men: >14 standard units/week  Inactive/low physical activity level: <4 hours of light exercise/week.  Missing values, n: urinary albumin excretion: 34, BMI: 1, abdominal fat distribution: 2 | | | | |


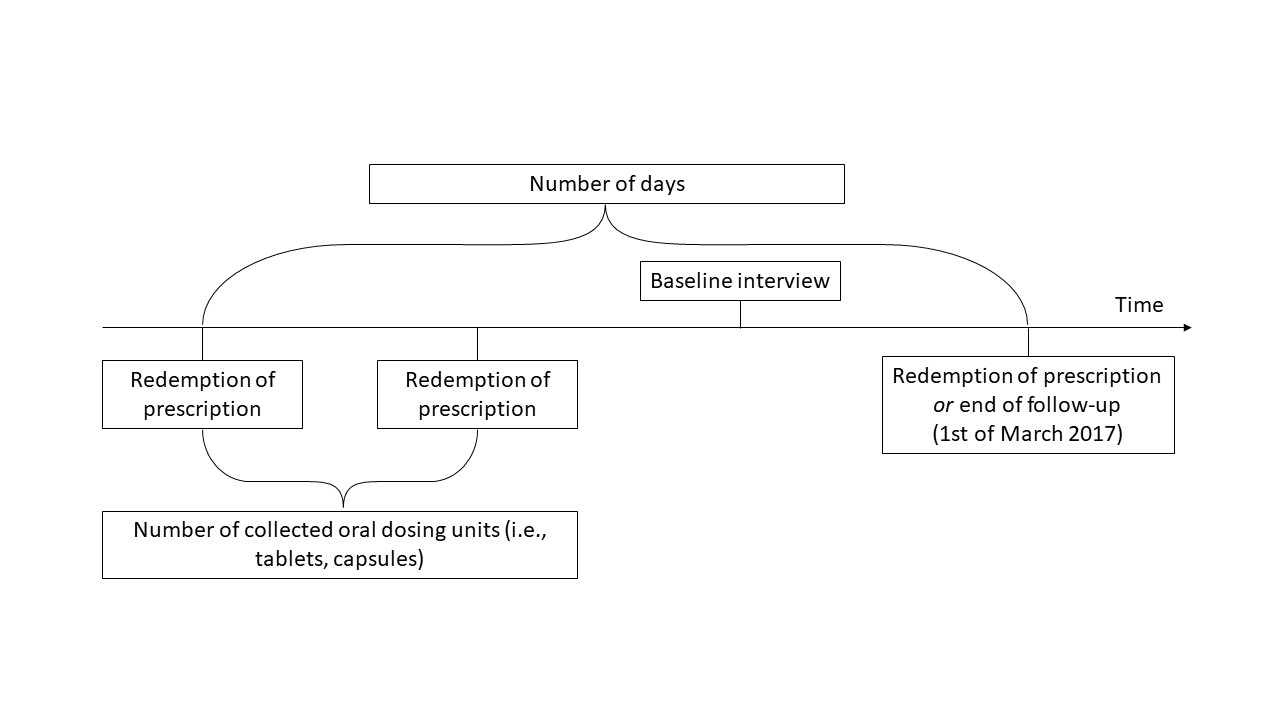


**Supplementary Figure S1: Estimation of medication possession ratio**

Medication possession ratio (MPR) calculated as the days’ supply collected divided by number of days between first and third prescription redemption with consideration of change of dose and discontinuation in this period. Omission of prescriptions categorized as “pro necessitate”, discontinued prescriptions (MPR<40% and patient reporting not taking the given medication, n=11), patients passing away before third redemption of prescription, patients with collection of days’ supply for ≥6 months, and patients with baseline interview <4 months before end of follow-up.
